# Prevalencia y factores asociados con la práctica de actividad física en mujeres gestantes adultas en Colombia

**DOI:** 10.7705/biomedica.6307

**Published:** 2022-06-01

**Authors:** Yuri Sánchez-Martínez, Diana Marina Camargo-Lemos, Myriam Ruiz-Rodríguez, Camilo A. Triana, Olga L. Sarmiento

**Affiliations:** 1 Departamento de Salud Pública, Escuela de Medicina, Facultad de Salud, Universidad Industrial de Santander, Bucaramanga, Colombia Universidad Industrial de Santander Universidad Industrial de Santander Bucaramanga Colombia; 2 Escuela de Fisioterapia, Facultad de Salud, Universidad Industrial de Santander, Bucaramanga, Colombia Universidad Industrial de Santander Universidad Industrial de Santander Bucaramanga Colombia; 3 Departamento de Salud Pública, Escuela de Medicina, Universidad de los Andes, Bogotá, D.C., Colombia Universidad de los Andes Universidad de los Andes Bogotá D.C Colombia

**Keywords:** embarazo, ejercicio físico, salud materna, actividades recreativas, encuestas y cuestionarios, salud pública, Pregnancy, exercise, maternal health, leisure activities, surveys and questionnaires, public health

## Abstract

**Introducción.:**

La actividad física durante el embarazo puede ser útil para la prevención de complicaciones gestacionales.

**Objetivo.:**

Estimar la prevalencia de actividad física en mujeres gestantes adultas en Colombia y evaluar los factores asociados con su práctica.

**Materiales y métodos.:**

Se hizo un análisis secundario de la información recolectada en la Encuesta Nacional de Situación Nutricional en Colombia del 2015. La muestra incluyó 906 mujeres gestantes. La actividad física en los dominios de tiempo libre, transporte y global se evaluó con la versión larga del International Physical Activity Questionnaire. Los factores asociados se evaluaron mediante modelos de regresión binomial negativa.

**Resultados.:**

La prevalencia del cumplimiento de las recomendaciones de actividad física en las participantes fue de 12,57 % (IC_95%_ 8,41-18,38), 28,66 % (IC_95%_ 23,29-34,70) y 36,33 % (IC_95%_ 30,92-42,11) en los dominios de tiempo libre, transporte y global, respectivamente. Los factores asociados con el dominio de tiempo libre fueron: residir en Bogotá (razón de prevalencia, RP=2,41; IC_95%_ 1,16-4,99), encontrarse en el tercer trimestre de la gestación (RP=2,13; IC_95%_ 1,17-3,87), disponer de programas de actividad física dirigida (RP=1,75; IC_95%_ 1,07-2,87), nivel educativo de secundaria (RP=0,51; IC_95%_ 0,29-0,91), y pertenecer a los cuartiles de riqueza dos (RP=0,45; IC_95%_ 0,24-0,81), tres y cuatro (RP=0,43; IC_95%_ 0,23-0,80). Los factores asociados con el transporte fueron: tener, por lo menos, un hijo (RP=1,60; IC_95%_ 1,14-2,24), residir en Bogotá (RP=1,84; IC_95%_ 1,23-2,73), convivir con compañero sentimental (RP=0,66: IC_95%_ 0,49-0,89) y haber asistido a entre uno y cuatro controles prenatales (RP=0,53; IC_95%_ 0,37-0,76).

**Conclusiones:**

La prevalencia de la actividad física en mujeres gestantes colombianas es preocupantemente baja. Se requiere la implementación de programas y proyectos orientados a la promoción de la actividad física durante el embarazo.

## Abstract (en)

**Introduction:**

Physical activity during pregnancy can be useful in the prevention of gestational complications.

**Objective:**

To estimate the prevalence of physical activity in adult Colombian pregnant women and its associated factors.

**Materials and methods:**

A secondary analysis of data from the 2015 National Nutrition Survey in Colombia was conducted. The total sample included 906 pregnant women. Leisure-time, commuting, and global physical activity were measured with the long version of the International Physical Activity Questionnaire. Factors associated with PA were evaluated through negative binomial regression models.

**Results:**

The prevalence of physical activity in pregnant women was 12.57% (95% CI=8.41- 18.38), 28.66% (95% CI=23.29-34.70), and 36.33% (95% CI=30.92-42.11) in the leisuretime, commuting and global domains, respectively. Factors associated with leisure-time physical activity were living in Bogotá (prevalence ratio, PR=2.41; 95% CI=1.16-4.99), being in the third gestational trimester (PR=2.13; 95% CI=1.17-3.87), obtaining access to physical activity programs (PR=1.75; 95% CI=1.07-2.87), having a high school education (PR=0.51; 95% CI=0.29-0.91), and being in the second (PR=0.45; 95% CI=0.24-0.81), third and fourth quartiles of wealth (PR=0.43; 95% CI=0.23-0.80). Factors associated with physical activity while commuting were having at least one child (PR=1.60; 95% CI=1.14-2.24), living in Bogotá (PR=1.84; 95% CI=1.23-2.73), living with a partner (PR=0.66; 95% CI=0.49-0.89), and completing between one and four prenatal care sessions (PR=0.53; 95% CI=0.37-0.76).

**Conclusions:**

The prevalence of physical activity in pregnant Colombian women is worryingly low. Programs and projects aimed at promoting physical activity during pregnancy are needed.

La práctica de actividad física regular para la población en general tiene múltiples beneficios para la salud y el bienestar ^1^. La Organización Mundial de la Salud (OMS) recomienda a las mujeres gestantes saludables que no presenten complicaciones durante el embarazo, acumular 150 minutos semanales de actividad moderada, la cual debe ser prescrita de manera individual ^1^. Quienes han sido físicamente activas antes del embarazo pueden continuar con sus actividades habituales, a menos que presenten alguna complicación obstétrica ^1^^,^^2^.

La prevalencia mundial de cumplimiento de recomendaciones sobre la actividad física es del 68 % en mujeres ^3^, en tanto que, en Colombia, es del 42,7 % ^4^. En mujeres gestantes se ha estimado en Estados Unidos una prevalencia de actividad física del 23,4 % entre el 2007 y el 2014 ^5^. En Latinoamérica, la información es limitada y no hay evidencia de datos nacionales. En ciudades de Brasil, el cumplimiento de estas recomendaciones en mujeres gestantes varía entre el 8,5 y el 12,9 % ^6^^,^^7^. En Colombia, en dos estudios en ciudades del norte del país, se encontraron prevalencias entre el 30,9 ^8^ y el 38 % ^9^.

El embarazo es un periodo crítico para el fomento de estilos de vida saludables como la práctica de actividad física por el interés de la madre en el bienestar propio y el de su hijo ^10^. Infortunadamente, durante la gestación se observa una reducción de los niveles de actividad física ^7^, con tendencia a continuar disminuyendo a medida que avanza la edad gestacional ^6^^,^^7^.

La evidencia sugiere que la práctica de actividad física durante el embarazo implica un riesgo mínimo para la salud materno-fetal ^2^ y, por el contrario, hay información sobre sus beneficios, como un menor riesgo de preeclampsia ^11^, diabetes gestacional ^12^, parto por cesárea ^13^ y parto prematuro ^10^, además de contribuir a un mejor control de la ganancia de peso durante el embarazo ^2^. Este último es particularmente importante debido a que se asocia con otras complicaciones como hipertensión arterial gestacional ^14^, prolongación del trabajo de parto y macrosomía fetal ^15^, con sobrepeso infantil a largo plazo ^16^.

También, se ha sugerido que los efectos positivos de la actividad física gestacional sobre la salud del hijo se extienden a la fase posnatal ^17^^-^^19^. Los hijos de madres físicamente activas durante el embarazo presentan mejores puntajes en las mediciones del neurodesarrollo ^17^ y el desempeño psicomotor a los 12 meses de edad ^18^, así como en las evaluaciones de inteligencia general y de habilidades de lenguaje a los 5 años ^19^, comparados con los hijos de las mujeres gestantes inactivas.

En la literatura revisada, se encontró que Colombia es el primer país de Latinoamérica en evaluar la actividad física en mujeres gestantes en una muestra con representatividad nacional mediante la Encuesta Nacional de la Situación Nutricional (ENSIN) en el 2015 ^4^, razón por la cual los resultados de este estudio son relevantes desde la perspectiva de salud pública, pues la caracterización de estos comportamientos y la determinación de sus factores asociados contribuirá al diseño de estrategias para la promoción de la actividad física durante la gestación, con los consiguientes beneficios para la salud de las mujeres gestantes y de sus hijos.

En este contexto, el objetivo de este estudio fue estimar la prevalencia del cumplimiento de las recomendaciones sobre actvidad física, y evaluar los factores asociados con su práctica en mujeres gestantes colombianas entre los 18 y los 48 años.

## Materiales y métodos

Se hizo un análisis secundario de la ENSIN 2015, la cual ha venido realizándose en Colombia cada cinco años desde el 2005. Esta es una encuesta de base poblacional, de corte transversal, con cobertura nacional y representatividad urbana y rural de seis regiones, 14 subregiones y 32 departamentos seleccionados mediante muestreo probabilístico de conglomerados estratificado y polietápico. Los criterios de inclusión de la ENSIN 2015 fueron pertenecer a la población civil no institucionalizada y ser residente habitual del territorio nacional.

La muestra final de la ENSIN 2015 estuvo compuesta por 44.202 hogares ubicados en 295 municipios de los 32 departamentos del país y en Bogotá. Se entrevistaron 1.140 mujeres gestantes entre los 18 y los 48 años. La información fue recolectada digitalmente entre noviembre del 2015 y diciembre del 2016.

### Actividad física

La actividad física se evaluó aplicando la versión larga del *International Physical Activity Questionnaire* (IPAQ), previamente validada y recomendada para su aplicación en Latinoamérica ^20^. Se evaluaron los dominios de tiempo libre, transporte y global (tiempo libre más transporte). La actividad física se midió como el tiempo en minutos dedicado a su práctica en cada uno de los dominios mencionados durante los siete días previos a la aplicación del cuestionario. Posteriormente, considerando lo propuesto por la OMS ^1^ y las indicaciones del manual IPAQ ^21^, se calculó el cumplimiento de las recomendaciones en cada dominio, definiendo como punto de corte 150 minutos semanales.

Se excluyeron de la evaluación a aquellas mujeres gestantes que respondieron afirmativamente a la siguiente pregunta “¿Su médico o un profesional de la salud le han recomendado no hacer actividad física debido a que su embarazo es de alto riesgo?” y a quienes, por defecto, tenían contraindicación para la práctica de actividad física. El total de participantes incluidas en nuestro análisis fue de 906.

### Variables sociodemográficas y del embarazo

Se recolectó información sobre: la edad en años cumplidos categorizada en dos grupos (18 a 25 y 26 a 48); el nivel educativo medido como el número de años escolares aprobados y categorizado como ninguno o primaria (0 a 5 años), secundaria (6 a 11 años) y superior (≥12 años); el cuartil de riqueza, correspondiendo el 1 al más pobre y el 4 al más rico; la convivencia con un compañero sentimental (sí o no) y la paridad (sí o no); la procedencia (rural o urbana), y la región geográfica, (Atlántico, Oriental, Orinoquía-Amazonía, Bogotá, Central o Pacífica). Además, se evaluaron características del embarazo como la edad gestacional categorizada por trimestres y el número de controles prenatales (0, 1-4 y >4), registrando si estos eran los esperados según la edad gestacional (sí o no).

### Variables contextuales

Durante la encuesta, se preguntó sobre la disponibilidad de espacios para la práctica de actividad física en el barrio o vereda (sí o no), de ciclovías (sí o no) y de programas de actividad física dirigida (sí o no). A quienes respondieron afirmativamente a cada una de estas preguntas, se les indagó si participaban o asistían a estos lugares o programas (sí o no).

Todos los protocolos fueron aprobados por el Comité de Ética de Profamilia.

### Análisis estadístico

El análisis estadístico siguió las siguientes fases: primero, se determinaron las prevalencias de cumplimiento de las recomendaciones sobre actividad física en el tiempo libre, transporte y global. En la segunda fase, estas prevalencias se estratificaron según las variables independientes: características sociodemográficas, del embarazo y contextuales. Las diferencias en las prevalencias de cada dominio, según las categorías de las variables independientes, se evaluaron con la prueba de ji al cuadrado. Por último, en la tercera fase, se aplicaron modelos de regresión binomial negativa para estimar las razones de prevalencia en la evaluación de los posibles factores asociados con tiempo dedicado a actividades físicas en el tiempo libre, transporte y global. Todos los análisis estadísticos se ajustaron según los pesos muestrales y el diseño de la muestra utilizando el programa Stata 13.

## Resultados

Después de excluir a las mujeres gestantes con contraindicación para la práctica de actividad física, la muestra final quedó conformada por 906 mujeres en quienes se usó el IPAQ.

La mediana de edad fue de 25 años (rango intercuartílico, RIC=21-29) y la de educación fue de 11 años (RIC=7-12). El 60,74 % de las participantes tenía, al menos, un hijo; el 35,60 % se encontraba en el primer trimestre gestacional y, el 36,53 %, en el segundo. El 62,14 % de ellas mencionó la existencia de espacios para la práctica de actividad física en su barrio o vereda y el 63,64 % informó que disponía de por lo menos un tipo de programa de actividad física en su ciudad o municipio; de este porcentaje solo el 5,44 % reportó haber asistido a uno de estos programas durante el mes anterior a la encuesta.

### Prevalencia de cumplimiento de las recomendaciones sobre actividad física

El cumplimiento de estas recomendaciones fue del 12,57 % (IC_95%_ 8,4118,38), el 28,66 % (IC_95%_ 23,29-34,70) y el 36,33 % (IC_95%_ 30,92-42,11) en los dominios de tiempo libre, transporte y global, respectivamente.

Las participantes con nivel educativo superior, pertenecientes a los cuartiles de riqueza tres y cuatro, residentes en zonas urbanas, con los controles prenatales esperados según la edad gestacional y la disponibilidad de espacios para la práctica de actividad física, cumplieron con mayor frecuencia las recomendaciones sobre actividad física en el tiempo libre, transporte y global.

Las residentes en zona urbana, pertenecientes a la región Pacífica y con disponibilidad de un programa dirigido de actividad física cerca de su residencia, reportaron un mayor cumplimento de las recomendaciones sobre actividad física en el tiempo libre, transporte y global. Por último, quienes refirieron no convivir con un compañero sentimental y disponer de un programa dirigido de actividad física cerca de su residencia, reportaron un mayor cumplimiento de las recomendaciones sobre actividad física en el tiempo libre y global (cuadro 1).

**Cuadro 1:** Cumplimiento de recomendaciones sobre actividad física según características sociodemográficas, del embarazo y contextuales, en mujeres gestantes colombianas entre los 18 y los 48 años. Encuesta Nacional de la Situación Nutricional en Colombia, 2015

		AF durante el tiempo libre^a^			AF como medio de transporte^b^			AF total^c^		
		n	%	^p^	n	%	^p^	n	%	^p^
Características sociodemográficas										
Edad (años)										
	18-25	39	10,5	0,383	119	119	0,856	158	35,9	0,867
	>25	32	14,79		95	95		126	36,78	
Nivel educativo										
	Ninguno o primaria	17	12,51	0,004	30	25,84	0,926	41	30,66	0,663
	Secundaria	31	6,47		116	28,86		153	35,96	
	Superior	22	21,28		65	29,43		86	38,98	
Cuartil de riqueza										
	1	40	9,13	0,030	94	24,95	0,415	135	33,46	0,435
	2	16	8,15		59	28,34		75	34,42	
	3 y 4	15	20,27		61	33,47		74	41,35	
Estado civil (convive con compañero)										
	Sí	55	12,37	0,844	148	27,06	0,258	205	33,09	0,020
	No	16	13,16		66	33,21		79	45,59	
Paridad (tiene hijos)										
	Sí	50	12,69	0,978	144	33,1	0,078	188	38,49	0,382
	No	19	12,56		66	22,69		90	33,49	
Procedencia									
	Urbana	53	14,51	0,023	167	31,33	0,049	222	38,42	0,119
	Rural	18	7,35		47	21,46		62	30,71	
Región										
	Atlántico	17	12,72	0,163	44	22,97	0,009	70	30,33	0,085
	Oriental		7,43		34	26,35		41	35,3	
	Orinoquía y Amazonía		5,07		28	16,99		43	28,62	
	Bogotá		25,17		26	57,9		26	57,9	
	Central		14,73		48	25,02		62	37,87	
	Pacífica		6,37		34	26,53		42	31,83	
Características del embarazo										
Edad gestacional (trimestre)										
	1	21	7,24	0,076	71	24,83	0,406	91	29,48	0,058
	2	25	11,81		75	28,37		100	35,9	
	3	25	20,4		68	33,92		93	45,64	
Controles prenatales realizados										
	0	11	5,68	0,178	48	32,89	0,518	55	36,16	0,135
	1 a 4	26	13,94		69	26,36		92	31,87	
	>4	24	18,24		68	32,46		96	44,37	
Controles según la edad gestacional										
	Sí	24	20,11	0,016	68	29,99	0,879	97	39,2	0,736
	No	35	8,39		111	30,93		142	37,18	
Características contextuales										
Disponibilidad de espacios para la práctica de AF										
	Sí	42	15,53	0,027	129	31,38	0,150	169	39,98	0,055
	No	29	7,73		85	24,19		115	30,33	
Seguridad en espacios para la práctica de AF										
	Sí	28	17,2	0,041	88	32,2	0,245	119	42,11	0,057
	No	43	8,58		126	25,6		165	31,33	
Disponibilidad de ciclovía										
	Sí	39	14,02	0,434	121	33,28	0,067	162	39,84	0,147
	No	32	10,77		93	22,87		122	31,94	
Disponibilidad de PAF dirigido										
	Sí	37	16,11	0,109	111	34,72	0,024	150	41,91	0,037
	No	34	9,31		103	23,07		134	31,18	

AF: actividad física; PAF: programa de actividad física

Se presentan proporciones por filas

Prueba: ji al cuadrado

^a^ Definida como la práctica de actividad física moderada en tiempo libre durante por lo menos 150 minutos en los últimos 7 días o realizar actividad física vigorosa por lo menos durante 75 minutos en los últimos 7 días, o una combinación equivalente de las dos

^b^ Definida como la práctica de actividad física como medio de transporte por lo menos durante 150 minutos en los últimos 7 días

^c^ Definida como la práctica de actividad física moderada a vigorosa en tiempo libre + actividad física como medio de transporte durante por lo menos 150 minutos en los últimos 7 días

### Factores asociados con el cumplimiento de las recomendaciones sobre actividad física

Se encontró que las mujeres gestantes residentes en Bogotá dedicaban 1,41 veces más tiempo en minutos a la práctica de actividad física de tiempo libre, transporte y global, comparadas con quienes vivían en la región Atlántica. Quienes se encontraban en el tercer trimestre gestacional dedicaban 1,13 veces más minutos a la actividad física de tiempo libre, transporte y global, comparadas con las que estaban en el primer trimestre. Por último, el disponer de programas dirigidos de actividad física se asoció con 75 % más de minutos dedicados a la práctica de actividades físicas de tiempo libre, transporte y global.

**Figura 1:**
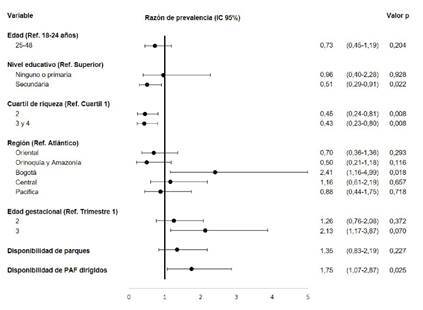
Factores asociados al tiempo dedicado a la actividad física durante el tiempo libre en mujeres gestantes colombianas entre los 18 y los 48 años.

En contraste, las mujeres gestantes con nivel educativo de secundaria reportaron 49 % menos minutos de actividades físicas de tiempo libre, transporte y global, comparadas con quienes tenían un nivel educativo superior, y aquellas pertenecientes a los cuartiles de riqueza 2 y 3 a 4 dedicaron 55 y 57 % menos tiempo a las actividades físicas de tiempo libre, transporte y global, respectivamente, comparadas con quienes se encontraban en el cuartil 1 de riqueza (figura 1).

Fuente: Encuesta Nacional de la Situación Nutricional en Colombia, 2015

**Figura 2:**
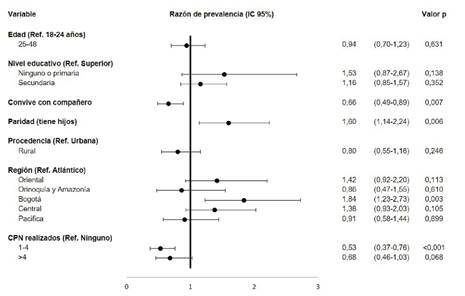
Factores asociados al tiempo dedicado a la actividad física como medio de transporte en mujeres gestantes colombianas entre los 18 y los 48 años. Encuesta Nacional de la Situación Nutricional en Colombia, 2015

Se encontró que las participantes que tenían un hijo, por lo menos, dedicaban 60 % más de minutos a las actividades físicas de tiempo libre y transporte, comparadas con quienes no tenían hijos. Asimismo, las residentes en Bogotá dedicaban un 84 % más de minutos a las actividades físicas de tiempo libre y transporte y, comparadas con quienes residían en la región Atlántica. En contraste, convivir con el compañero sentimental se asoció con un 34 % menos de tiempo dedicado a las actividades físicas de tiempo libre y transporte (figura 2).

Fuente: Encuesta Nacional de la Situación Nutricional en Colombia, 2015

**Figura 3:**
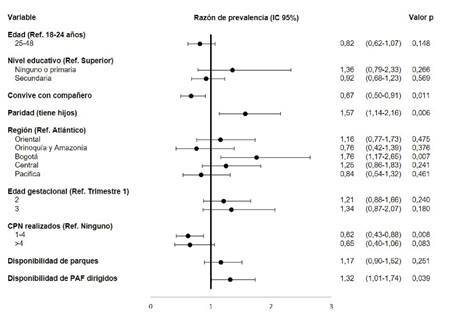
Factores asociados al tiempo dedicado a la actividad física global en mujeres gestantes colombianas entre los 18 y los 48 años. Encuesta Nacional de la Situación Nutricional en Colombia, 2015

Los factores asociados con las actividades físicas de tiempo libre y global fueron similares a los encontrados en el dominio de transporte; además, se encontró una asociación positiva con la disponibilidad de programas dirigidos de actividad física (figura 3).

Fuente: Encuesta Nacional de la Situación Nutricional en Colombia, 2015

## Discusión

Este es el primer estudio en Latinoamérica en el que se evalúan los niveles de actividad física en una muestra nacional representativa de mujeres gestantes. Nuestros resultados evidenciaron que solo el 12,57 % de ellas estuvieron físicamente activas durante el tiempo libre; el 28,66 % lo estuvieron en el dominio de transporte y, el 36,33 %, de manera global. Es importante destacar la baja prevalencia de las actividades físicas de tiempo libre comparada con las de transporte, especialmente si se tiene en cuenta que la primera está asociada con mejores niveles de salud física y mental.

### Actividad física durante el tiempo libre

Los resultados de la ENSIN 2015 mostraron que solo 12 de cada 100 mujeres gestantes colombianas cumplían con los 150 minutos semanales de las actividades físicas de tiempo libre, dominio que en el 2015 en Estados Unidos registró una prevalencia del 23,4 % ^5^. Esta diferencia podría obedecer a las condiciones socioculturales de las mujeres gestantes colombianas o al tipo de pregunta en cada estudio. En la ENSIN 2015 se indagó por la actividad física durante los siete días previos a la encuesta, en tanto que en Estados Unidos se preguntó sobre la actividad física en una semana promedio, situación que puede llevar a una sobreestimación en el reporte.

Otra posible explicación de esta diferencia es la conformación étnica de la muestra de Estados Unidos, factor que previamente se ha asociado con las actividades físicas de tiempo libre ^22^. En ciudades como Johannesburgo (Sudáfrica) ^23^ y Bristol (Inglaterra) ^24^, se han informado prevalencias entre el 5 y el 48,8 %, destacándose esta última, lo que podría explicarse también por el tipo de pregunta utilizada, pues en este caso se indagó por, al menos, un día de práctica de actividad física durante la semana anterior; este tipo de medición puede sobreestimar el resultado si se lo compara con el empleado en el IPAQ. Es importante destacar que, a pesar de los beneficios potenciales de la actividad física en el embarazo, el cumplimiento de las recomendaciones sigue siendo muy bajo.

Además, la prevalencia de las actividades físicas de tiempo libre en mujeres gestantes fue menor a la reportada por mujeres no embarazadas (16,4 %) en la ENSIN 2015 ^4^. Esto coincide con lo ya reportado en la literatura especializada sobre el embarazo como un periodo de disminución en los niveles de actividad física ^7^. En nuestro contexto, esta menor frecuencia de actividad física en las mujeres gestantes que en las no embarazadas, podría estar influenciada por la falta de promoción y orientación de su práctica en los programas de control prenatal. En un estudio reciente sobre dichos programas en la red pública de Bucaramanga (Colombia), se encontró que solo el 26,1 % de las mujeres gestantes asistentes a dichos programas recibía recomendaciones para la práctica de actividad física ^25^, lo que resalta la importancia de intervenir en ellos, y reforzar la educación y preparación de los profesionales a cargo del cuidado de la salud materno-fetal, con el fin de brindar una orientación adecuada a las mujeres gestantes.

Se encontraron asociaciones negativas entre el tiempo dedicado a las actividades físicas de tiempo libre y variables como el nivel educativo de secundaria comparado con el de educación superior, y los cuartiles de riqueza más altos comparados con el cuartil más bajo. En cuanto al nivel educativo, nuestros hallazgos coinciden con los artículos revisados, en los que se ha establecido una mayor actividad física de tiempo libre en los grupos con mayor escolaridad ^6^^,^^26^, lo que podría obedecer a una mejor percepción de sus beneficios para la salud entre las embarazadas con mayor escolaridad.

En cuanto a la asociación entre el cuartil de riqueza y la las actividades físicas de tiempo libre, nuestros resultados son contrarios a los reportados en estudios previos ^6^^,^^27^, pues los datos de la Encuesta Nacional de Demografía y Salud en Colombia 2015 evidenciaron una mayor prevalencia de mujeres que trabajaban en los quintiles más altos de riqueza ^28^, y podríamos suponer que un mayor nivel de ocupación laboral implica menos tiempo libre para la práctica de actividades físicas. Otra explicación podría plantearse desde el punto de vista metodológico. En este sentido, Domingues, *et al*. ^6^, abordaron a las madres en las 24 horas posteriores al parto y les preguntaron sobre la práctica de algún tipo de actividad física en cada trimestre, lo que podría haber generado un sesgo de recuerdo y sobreestimaría el tiempo dedicado a la actividad física por tratarse de un comportamiento socialmente deseable.

Nuestros hallazgos mostraron que residir en Bogotá, estar en el tercer trimestre de embarazo y disponer de programas de actividad física dirigidos en el barrio o vereda, se asociaron con un mayor tiempo dedicado a la práctica de las actividades físicas de tiempo libre.

Con base en la literatura revisada, puede afirmarse que este es el primer estudio en Colombia en el que se evalúa la asociación entre la actividad física y la región geográfica de residencia, por lo tanto, no fue posible comparar nuestros hallazgos con otros estudios. La asociación positiva encontrada entre las actividades físicas de tiempo libre y residir en Bogotá, podría explicarse por la gran oferta de programas y servicios relacionados con actividad física en esta ciudad y en Colombia, donde el programa Hábitos y Estilos de Vida Saludables del Ministerio del Deporte llega a los 32 departamentos del país ^29^. Bogotá, bajo la coordinación del Instituto Distrital de Recreación y Deporte, cuenta con la ciclovía-recreovía, reconocida como la de mayor extensión en el mundo ^30^, la cual destaca a la capital colombiana en el ámbito mundial.

En cuanto a la asociación positiva entre la disponibilidad de programas de actividad física dirigida en el municipio o vereda de residencia y las actividades físicas de tiempo libre, se encontraron estudios realizados en la población general en Bogotá y Vitoria (Brasil), con resultados similares a los nuestros ^26^^,^^31^. Asimismo, se ha reportado que, cuando existen tales programas, son las mujeres quienes asisten en mayor proporción ^26^ y que el hecho de participar en ellos, se asocia con un estilo de vida activo, que se mantiene inclusive después de dejar de asistir ^32^.

La asociación positiva entre el tercer trimestre gestacional y las actividades físicas de tiempo libre podría obedecer a que, después del primer trimestre, los síntomas como náuseas, fatiga, somnolencia y astenia empiezan a disminuir, así como el temor a un aborto espontáneo, lo que favorecería el restablecimiento de actividades diarias como las caminatas, reportadas como el tipo de actividad física más frecuente durante el embarazo ^7^. Aunque la ENSIN 2015 no cuenta con información sobre el tipo de actividad física de las mujeres gestantes, es evidente una mayor prevalencia de desplazamientos activos a medida que aumenta la edad gestacional, observándose un incremento importante entre el segundo y tercer trimestres, lo cual sugiere que las caminatas se harían como preparación para el parto debido a su bajo impacto y fácil ejecución ^7^.

Por otro lado, en los modelos ecológicos propuestos por Sallis, *et al*. ^33^, se destaca la importancia de los espacios públicos para la promoción de la actividad física. En un estudio en mujeres gestantes de Estados Unidos, se encontró que disponer de gimnasios y áreas recreativas cerca de casa se asoció con un mayor cumplimiento de las recomendaciones sobre actividad física en este grupo poblacional ^34^. Aunque nuestros resultados mostraron una asociación positiva entre la disponibilidad de espacios para la práctica de actividad física en el barrio o la vereda y la práctica de actividades físicas de tiempo libre, esta no fue estadísticamente significativa; sin embargo, hay un acuerdo mundial sobre la importancia de los espacios públicos y de los programas dirigidos a todos los grupos poblacionales como parte del Plan de Acción Global sobre Actividad Física, 2018-2030, propuesto por la OMS ^35^.

### Actividad física en el dominio de transporte y en el global

El análisis multivariado de los dominios de transporte y global arrojó resultados similares, por lo que se discuten de manera conjunta. La ENSIN 2015 registró que convivir con un compañero y haber completado entre uno y cuatro controles prenatales, se asociaron negativamente con las actividades físicas de transporte y global, hallazgos de difícil comparación con otros estudios por la escasa literatura disponible sobre la actividad física como medio de transporte. Watson, *et al*. ^23^, también encontraron una asociación negativa con la convivencia con un compañero, explicada por un posible doble ingreso en el hogar, lo que favorecería la disponibilidad de un vehículo familiar y disminuiría el uso del transporte activo de los integrantes de la familia.

Llama la atención la asociación negativa entre el tiempo de A actividades físicas de transporte y el número de controles prenatales, hallazgo que no había sido reportado previamente en ningún estudio. Sin embargo, es posible que las mujeres gestantes opten por medios de transporte motorizados para el cumplimiento de las citas programadas. De todas maneras, la relación entre los controles prenatales y la actividad física requiere mayor estudio; además, los profesionales de la salud deben capacitarse para su promoción.

Entre los factores asociados positivamente con la actividad física de transporte y la global, se determinaron la residencia en Bogotá y tener por lo menos un hijo. Como se mencionó previamente, no se encontró información en los estudios revisados que permitiera comparar nuestros hallazgos. Sin embargo, el mayor transporte activo en Bogotá, en comparación con la región de referencia, podría deberse a la gran congestión vehicular en la capital colombiana. Esto, sumado a encontrarse embarazada y tener más de un hijo, dificultaría el desplazamiento de las mujeres gestantes en el sistema de transporte masivo de la ciudad, lo que podría favorecer la caminata como medio de transporte.

### Implicaciones en política pública

Nuestros hallazgos mostraron una mayor práctica de actividad física por parte de las mujeres gestantes residentes en lugares con políticas de mayor trayectoria en este campo, así como programas que se desarrollan de manera regular y están orientados a la promoción de dicho comportamiento. Por ello, consideramos importante que dichas políticas y programas incluyan a las mujeres gestantes como parte de su población objetivo, considerando sus necesidades particulares. Así, tanto ellas como sus hijos podrían obtener los beneficios para la salud derivados de la práctica de la actividad física que, como se ha reportado previamente, pueden extenderse incluso después del parto ^17^^-^^19^. También, es necesaria la promoción de la actividad física en las mujeres en edad fértil, pues ello facilitaría que se mantuvieran físicamente activas en caso de quedar embarazadas. Algunos estudios han reportado que las mujeres físicamente activas antes del embarazo tienen una mayor probabilidad de continuar siéndolo durante la gestación ^7^.

La baja prevalencia de actividad física en las mujeres gestantes colombianas plantea la necesidad de involucrar a las asociaciones de ginecología y obstetricia, así como a los responsables de las decisiones, para que establezcan guías de recomendaciones de actividad física durante el embarazo como una política de salud pública, con la participación de equipos multidisciplinarios capacitados que sirvan de apoyo a los programas de control prenatal en la promoción de la actividad física durante el embarazo y orienten a las mujeres gestantes en su práctica, con el fin de prevenir los problemas de salud de madres e hijos asociados con el sedentarismo y la obesidad.

### Fortalezas y limitaciones

Entre las fortalezas de nuestro estudio cabe destacar, nuevamente, que es el primero en Latinoamerica que ofrece información sobre los niveles de actividad física en mujeres gestantes y sus factores asociados en una muestra con representación nacional. Además, la actividad física se midió mediante el IPAQ, un cuestionario validado y recomendado ^20^ ampliamente usado, lo que permite disponer de información comparable con otros estudios. Por otra parte, la aplicación de modelos multivariados en el análisis permitió controlar un potencial sesgo de confusión.

No obstante, como todo estudio también tuvo limitaciones, entre otras, la posibilidad del sesgo de información derivado de la medición mediante cuestionarios, a pesar de sus propiedades psicométricas ya demostradas ^20^ y el adecuado entrenamiento del personal encargado de recolectar la información. El 20,03 % de las participantes manifestaron tener contraindicada la actividad física, porcentaje que es mayor a lo reportado internacionalmente ^6^^,^^7^ y limitó el tamaño de la muestra.

Es claro que, a pesar de la evidencia sobre los beneficios de la práctica de la actividad física durante la gestación, su prevalencia en Colombia es muy baja. Los resultados de este estudio ofrecen evidencia científica sobre este fenómeno y representan una oportunidad para la adopción de políticas, programas y proyectos dirigidos a su fomento. Se requieren más estudios que permitan fortalecer el conocimiento en este tema, con el fin de facilitar la intervención sobre los estilos de vida durante el embarazo.
